# Image-guided Raman spectroscopy navigation system to improve transperineal prostate cancer detection. Part 1: Raman spectroscopy fiber-optics system and *in situ* tissue characterization

**DOI:** 10.1117/1.JBO.27.9.095003

**Published:** 2022-09-01

**Authors:** Fabien Picot, Roozbeh Shams, Frédérick Dallaire, Guillaume Sheehy, Tran Trang, David Grajales, Mirela Birlea, Dominique Trudel, Cynthia Ménard, Samuel Kadoury, Frédéric Leblond

**Affiliations:** a Polytechnique Montréal, Department of Engineering Physics, Montreal, Quebec, Canada; b Centre de recherche du Centre hospitalier de l’Université de Montréal, Montreal, Quebec, Canada; c Polytechnique Montréal, Medical Laboratory, Montreal, Quebec, Canada; d Institut du cancer de Montréal, Montreal, Quebec, Canada

**Keywords:** Raman spectroscopy, prostate cancer, tissue optics, multimodal imaging, machine learning, support vector machines, magnetic resonance imaging, ultrasound imaging

## Abstract

**Significance:**

The diagnosis of prostate cancer (PCa) and focal treatment by brachytherapy are limited by the lack of precise intraoperative information to target tumors during biopsy collection and radiation seed placement. Image-guidance techniques could improve the safety and diagnostic yield of biopsy collection as well as increase the efficacy of radiotherapy.

**Aim:**

To estimate the accuracy of PCa detection using *in situ* Raman spectroscopy (RS) in a pilot in-human clinical study and assess biochemical differences between *in vivo* and *ex vivo* measurements.

**Approach:**

A new miniature RS fiber-optics system equipped with an electromagnetic (EM) tracker was guided by trans-rectal ultrasound-guided imaging, fused with preoperative magnetic resonance imaging to acquire 49 spectra *in situ* (*in vivo*) from 18 PCa patients. In addition, 179 spectra were acquired *ex vivo* in fresh prostate samples from 14 patients who underwent radical prostatectomy. Two machine-learning models were trained to discriminate cancer from normal prostate tissue from both *in situ* and *ex vivo* datasets.

**Results:**

A support vector machine (SVM) model was trained on the *in situ* dataset and its performance was evaluated using leave-one-patient-out cross validation from 28 normal prostate measurements and 21 in-tumor measurements. The model performed at 86% sensitivity and 72% specificity. Similarly, an SVM model was trained with the *ex vivo* dataset from 152 normal prostate measurements and 27 tumor measurements showing reduced cancer detection performance mostly attributable to spatial registration inaccuracies between probe measurements and histology assessment. A qualitative comparison between *in situ* and *ex vivo* measurements demonstrated a one-to-one correspondence and similar ratios between the main Raman bands (e.g., amide I-II bands, phenylalanine).

**Conclusions:**

PCa detection can be achieved using RS and machine learning models for image-guidance applications using *in situ* measurements during prostate biopsy procedures.

## 1 Introduction

In 2020, 1.4 million cases of prostate cancer (PCa) were diagnosed worldwide with an estimated 375,000 deaths.^1^ In the United States, PCa is the most common and second deadliest cancer for men, accounting for 11% of total cancer deaths.^2^ Trans-rectal ultrasound-guided (TRUS) biopsy procedure is the standard of care for diagnosis of PCa due to its real-time capabilities and convenience.^3^^–^^5^ However, the genotypic and phenotypic heterogeneity of prostate tumors presents challenges in validating their position, type, extent, and grade to establish the diagnosis and prognosis.^6^^,^^7^ As a result, a significant rate of false negatives is reported for prostate biopsy procedures, reaching up to 30%.^8^^,^^9^

To address this clinical problem, electromagnetic (EM) tracking has helped to enhance the guidance of brachytherapy procedures^10^^–^^12^ and may have potential to guide prostate biopsy surgery. Furthermore, magnetic resonance imaging (MRI) navigation systems demonstrated superior sensitivity and equivalent or superior specificity to TRUS-guided biopsy,^13^^,^^14^ including an 85% sensitivity for prostate tumors larger than 10 mm.^15^ The limited access and higher costs of MRI for intraoperative and real-time surgical use^16^^,^^17^ has incentivized the use of MRI-TRUS fusion technology, which combines the high sensitivity of MRI and the high specificity of TRUS while removing the intraoperative use of MRI. By relying on pre-operative MRI images, MRI-TRUS fusion technology can further increase the accuracy of the biopsy procedure. However, due to the dynamic tissue deformation caused by the surgical instrument, the image registration between these modalities remains a challenge.^18^^–^^20^

Raman spectroscopy (RS) is a technique that can assess the biomolecular content of a sample through its optical properties. RS collects information about the vibrational states, which are specific to the molecules involved in the Raman scattering events. The molecular information infers differences in tissues, providing a real-time confirmation of tumor location during therapy. In the last decades, RS has been used for the *in vivo* diagnosis of several cancer types. Multiple research groups have successfully targeted skin cancers in open surgeries.^21^^–^^23^ Mcgregor et al. used Raman probes for a minimally invasive diagnosis of peripheral and early lung cancers.^24^^,^^25^ The state-of-the-art technology has also combined fingerprint (FP) and high wavenumber (HWN) spectral domains to further increase the sensitivity and specificity of Raman systems, notably on oral, colon, and brain cancers.^26^^–^^28^ Aubertin et al. conducted two *ex vivo* studies on human prostate, resulting in classification models that discriminated cancer from normal tissue with >85% sensitivity and specificity.^29^^,^^30^ Since 2019, similar performances have been reported in an *ex vivo* Raman microscopy study,^31^ whereas a Raman probe integrated to a robotic-assisted surgical system allowed the *in vivo* collection of extraprostatic and prostatic preliminary spectra without classification.^32^ To the best of our knowledge, there is no in-human study reporting the use of a Raman system for the purpose of PCa diagnosis.

We conducted a pilot clinical study to evaluate the feasibility and performance of a multimodal system, combining RS and MRI-TRUS guidance using EM tracking, for PCa diagnosis during surgery. For clarity and conciseness, we divided this study into two parts. This paper is Part 1 and presents our combined *ex vivo* and first in-human *in situ* RS study on prostate tissues from a total of 32 patients. An RS system in which an EM tracker was integrated was used to perform MRI-TRUS procedures on 18 patients. The 49 *in situ* spectra acquired from these patients were used as a training dataset to build a support vector machine (SVM) classification model discriminating cancer from normal prostate tissue. In addition, 179 *ex vivo* Raman measurements acquired from fresh prostate specimens of 14 patients who underwent radical prostatectomy (RP) were also used to further analyze the biomolecular content of prostate tissue and perform a qualitative comparison with the *in vivo* measurements. Part 2 of the study^33^ focuses on machine learning applications demonstrating that combining biomolecular characteristics from RS with imaging features from preoperative multiparametric MRI improves PCa detection accuracy.

## 2 Methods

### 2.1 Raman System and Calibration Processing

**Fig. 1:**
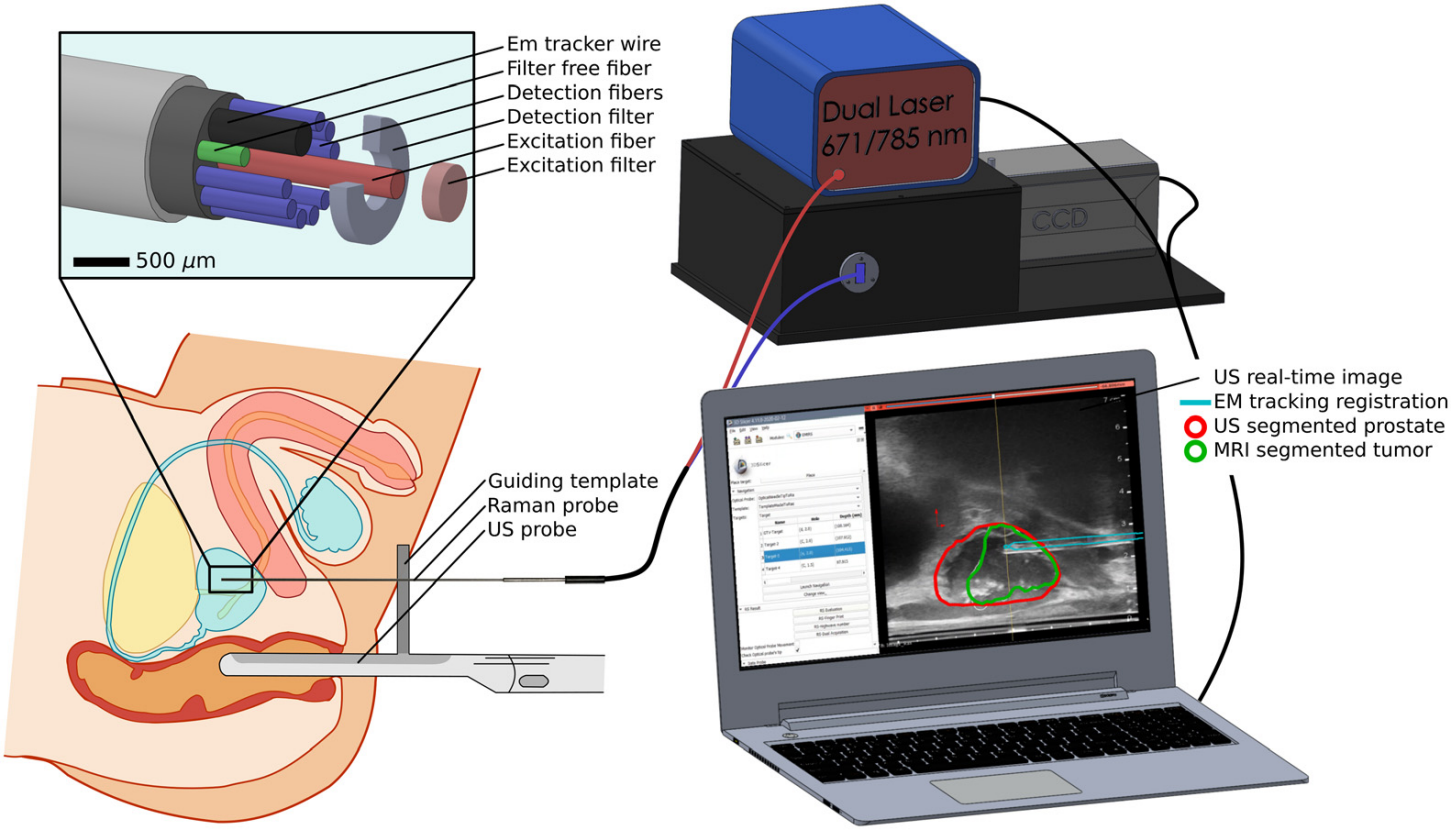
Schematic representation of the Raman probe obtaining measurements of the prostate through the guiding template, with a magnified view of the probe’s tip. The probe is connected to the laser source and the spectrometer, which are controlled by a computer, and the optical system is combined with a TRUS system to perform the prostate biopsy procedure through the surgical guiding template. The computer displays the fused TRUS-MRI guiding image and the raw optical spectra after spectral acquisition for each site.

Optical measurements were obtained using an RS fiber-optics probe (EmVision LLC, Florida) equipped with an EM tracker that was developed by our group for prostate biopsy applications (Fig. 1).^34^ The probe integrates 11 optical fibers and the EM tracker can sense six degrees of freedom (NDI Inc., Canada). The rigid part of the probe has a length of 20 cm and an outer diameter of 1.22 mm. The wire of the tracker is located 3.25 mm from the distal end of the probe next to the 10 fiber-optics collection fibers (100-μm-diameter core). Of these fibers, nine were intended to be used for Raman signal detection and one for fluorescence signal, and sensors encircled the length of the one Raman excitation fiber. However, no fluorescence measurements were made as part of this study. As shown in the magnified view of the probe tip in Fig. 1, two different filters are positioned to cover both collection and excitation Raman fibers. A band-pass filter, centered at 709 nm with full-width-at-half maximum (FWHM) of 172 nm (Semrock, New York), is placed at the end of the Raman excitation fiber to allow illumination at 671 and 785 nm for HWN and FP detection, respectively. A notch filter, centered at 785 nm with FWHM of 39 nm (Semrock, New York), is placed at the end of Raman collection fibers to prevent silicon signature from interfering with the signal of interest when using an excitation of 785 nm. Finally, one collection fiber is left without any filter to allow fluorescence signal acquisition although it is not used in the clinical protocol and was included in the design only for a future fluorescence system integration. A nylon heat shrink tubing seals and protects the probe’s tip. The probe itself is then connected to a dual-wavelength laser 671/785  nm (Innovative Photonics Solution, New Jersey) and a high sensitivity spectrometer at wavelengths from 800 to 900 nm, with an average resolution of $1.8\;{\mathrm{cm}}^{-1}$ (EmVision LLC, Florida). Two slightly different versions of this probe were used for our study; both were identical except for one probe that had a two-component lens at the probe’s tip to enhance signal collection and used of a 200-μm (instead of 100  μm) diameter core for the excitation fiber. The overall system was controlled by a Matlab (Mathworks) customized software to set acquisitions parameters: laser power P, exposure time per spectrum T, and number of repeated measurements n at each point. Complementary calibration data were acquired prior to experiments for normalization purpose. This included a dark count acquisition with illumination off, acetaminophen measurements (DiN 00789801, Trianon Inc., Canada) for x-axis calibration, and measurements of a Raman standard (NIST2241, NIST) for relative intensity calibration.

### 2.2 Navigation System

The navigation system used MRI-TRUS fusion guidance and is fully described in our previous study^34^ as well as in the companion study of this paper.^33^ Targets for the biopsy procedure were determined on preoperative MRI identification of regions of interest: based on multiparametric MRI, performed without contrast agent, the physician segmented the tumor and selected at least one biopsy site in the tumor and at least one biopsy site more than 10 mm away from the tumor. We used prototype interventional system (Invivo/UroNav, Philips Disease Management Solutions, Gainesville) to perform the fusion between MRI images and three-dimensional (3D) TRUS acquired with a BK3000 ultrasound and a BK Endocavity Biplane 8848 transducer (BK Medical, Denmark). The multiple subsystems, EM tracking, MRI-segmented images, and US were connected and coordinated using a customized 3D Slicer^35^ module allowing visualization of the registration. The navigation system was used during the prostate biopsy procedure to provide the surgeon with real-time anatomical guidance for instrument insertion, including the Raman probe, biopsy gun, and guiding cannulas. Only TRUS-MRI fused structures, without the RS, were used for navigation to the targets. They were only used for tissue classification purposes in Part 2 of the study.

### 2.3 Patient Selection

Informed consent was obtained from the 32 patients included in this study. Fourteen patients underwent prostate biopsy procedures revealing more than two PCa positive biopsy cores with a cancer involvement >10% for each core. Patients agreed to participate in the institutional PCa repository of the Centre hospitalier de l’Université de Montréal (CHUM Ethics Committees approval number 15.102) and underwent RP, leading to the exclusion of prostate specimens <35  g. Samples from these 14 patients were used to build the *ex vivo* dataset combining RS and histopathological data. Similarly, 18 patients were recruited in the study to acquire *in situ* data to build the *in vivo* dataset. Informed consents were obtained as well as agreement to participate in the institutional PCa repository before the patient underwent prostate biopsy procedure (CHUM Ethics Committees approval number 18.295). Clinical data were added to include age and preoperative prostate-specific antigen (PSA) of patients for both datasets. We also added the grade group (GG) from the whole prostate pathology result after RP and from the core needle biopsy procedure to the *ex vivo* and *in vivo* datasets, respectively. Finally, pathological stage (pT) was determined after RP procedures by a pathologist at the CHUM according to the TNM staging system, seventh edition.^36^ The details of these histopathological analyses are shown in Table 1.

**Table 1:** Clinical and pathological characteristics of the patients at RP and at biopsy procedure.

Characteristics	*Ex vivo*	*In vivo*
#patients (# measurements)	14 (179)	18 (49)
Median age	64 (62 to 67)	67 (65 to 70)
Median PSA (μg/μL)	5.5.6 (4.11 to 8.06)	5.84 (4.59 to 11.44)
(# measurements (cancer/normal)	27/152	21/28
PCa grade # patients		
0	0	3
1	1	1
2	6	6
3	5	5
4	0	3
5	2	0
Pathological tumor stage # patients		
pT2 (organ-confined)	8 (20)	—
pT3a (extraprostate extension)	4 (5)	—
PT3b (seminal vesicle extension)	2 (2)	—

The recruitment of the 32 patients led to two independent datasets: *ex vivo* and *in vivo*. Each dataset was used to build a classification model to assess the potential of RS to discriminate cancer from normal tissue and allowed a qualitative assessment of the main spectral differences between *in vivo* and *ex vivo* measurements.

### 2.4 Specimen Handling and *Ex Vivo* Measurements

**Fig. 2:**
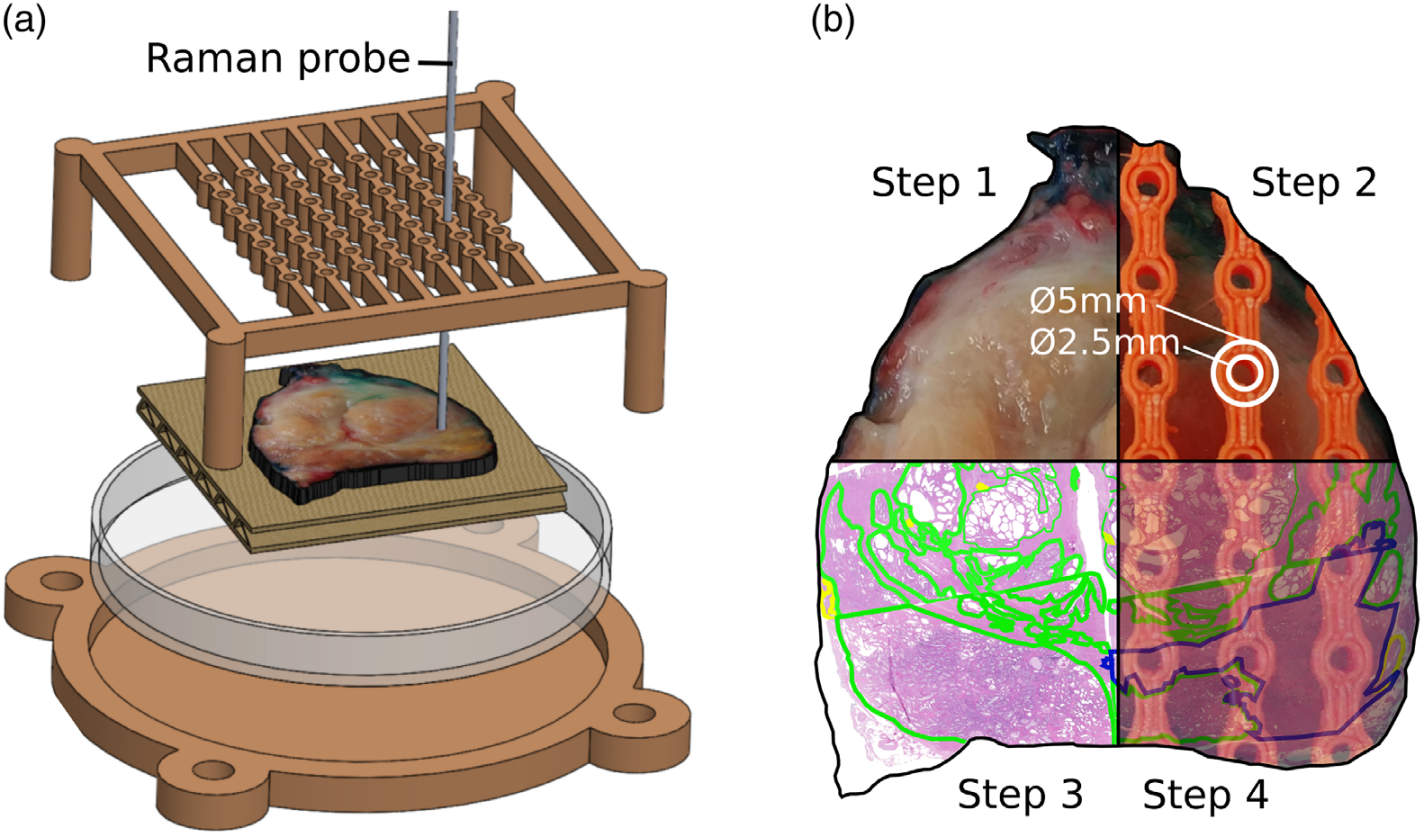
(a) Magnified view of the fresh prostate slice inside the guiding template for incremental Raman point measurements. (b) Four-step methodology for spatial registration of Raman measurements with labeled HPS tissue image reconstruction. Step 1 is a photograph of fresh prostate specimen; step 2 is a photograph of the fresh prostate specimen through guiding template; step 3 is HPS-labeled prostate image (cancer and normal tissue are labeled in blue and green, respectively); and step 4 is the superimposed images of step 2 and step 3.

Immediately after RP, the entire organ was weighed, measured, and marked with ink, following institutional standards.^37^ The prostate was cut in transverse plane perpendicular to the urethra, in ∼10 slices with a thickness of 3 to 5 mm each. The slice with the highest probability of containing a large cancer area, according to the biopsy pathology report, was removed and held between two pieces of waxed cardboard and then placed in the guiding template, as shown in Fig. 2(a). Raman measurements were taken sequentially through the guiding holes. The laser power P was optimized and set at a maximum of 20 to 50 mW to prevent tissue or instrument damage. Number of repeat measurements per point n was fixed at 50 and the exposure time per spectra T (0.1<T<2  s) was automatically adjusted and optimized to ensure a raw signal intensity between 85% and 100% of the camera dynamic range. After the measurements were done, the slice was put in 10% formalin and underwent standard histopathological analysis based on microscopic hematoxylin, phloxine, and saffron staining (HPS). In the time between its removal from the patient and its histopathological analysis, the prostate slice was prepared and measurements were acquired within a 2-h limit to ensure preservation of the sample. This time constraint puts a limit of an hour for the total optical acquisition time, allowing 5–20 point measurements per sample.

### 2.5 Prostate Slice Reconstruction and Registration with Optical Measurements

The process of registration between Raman measurement locations and HPS prostate images that assessed tissue as cancer or normal involved four steps. The first and second steps consisted of macroscopic photographs of the prostate slice in actual image and through the guiding template, respectively, as shown in Fig. 2(b). A contour was extracted from the image of stage 1 and served as a guiding boundary to reconstruct the HPS prostate image. Due to standard histopathological processing, the fresh prostate slice was segmented into 4 to 10 equal sections before staining. These sections were then imaged with an Aperio Digital Pathology Slide Scanner (Leica Biosystems, Nussloch, Germany) and were labeled by a pathologist to discriminate cancer area from normal tissue area. Stage 3 juxtaposed the labeled sections to build the complete HPS prostate image. For every section, multiple anatomical landmarks were used to ensure continuity between each other for accurate reconstruction. This included boundaries of cancerous nodules, ejaculatory ducts, prostatic urethra, and benign prostate hypertrophic nodules. Finally, stage 4 superimposed the results of the macroscopic fresh prostate photograph through the template from stage 2 and the HPS reconstructed prostate image from stage 3. Figure 2(b) shows for stage 4, every possible location of the probe through guiding template registered with a normal/cancer diagnosis.

Reconstruction of prostate slices and registrations with optical measurements were done using Inkscape software (Inkscape’s Contributors). Several factors could potentially limit the accuracy of the results, such as anisotropic tissue warping and uneven tissue level between adjacent sections. To mitigate the amount of classification error by our histopathological gold standard, we applied a spatial margin error. A measurement was labeled as normal if no cancer tissue was found in a 2.5 mm radius of the point localization. Similarly, a measurement was labeled as cancer and as cancer border if no normal tissue was found in a 2.5- and 1.25-mm radii, respectively, of the point localization. The choice to ensure a higher level of certainty for normal points than for cancer points was made because of the imbalance between these classes in the dataset, with only <15% of points labeled as cancer or cancer border.

### 2.6 Surgical Workflow and *In Vivo* Measurements

Once patients are under anesthesia, the lead surgeon sets up the US system to acquire a full 3D image of the prostate and its surrounding tissue and then operates the imaging system in real-time two-dimensional (2D) mode. The prostate is manually fully segmented in the 3D TRUS image and serves as a reference to register the imported 3D preoperative MRI data including the tumor segmentations. Although this step can be managed through the 3D slicer module, it is done by the *in vivo*/Uronav system fusion methods for workflow efficiency. The EM generator field is positioned by mechanical arm above the patient’s abdomen for the entire procedure. Two to four targets are defined on the MRI-TRUS navigation system and visualized on the augmented 2D real-time TRUS image. They are used by the surgeon to guide the insertion of cannulas to each biopsy target. The optical probe is inserted through the guiding cannulas and, with the probe’s tip making contact with the tissue, the Raman measurement is acquired (P=50  mW, n=50 or 100 and T optimized as exactly described in Sec. 2.4). The probe is then immediately removed and replaced by the prostate biopsy gun to ensure an exact spatial registration between the optical measurement and the sample’s location. A horizontal cross-sectional sample is extracted and the process is repeated for every target defined by the navigation system. The samples undergo a postoperative histopathological analysis (HPS staining) to provide a normal versus cancer label to build the *in vivo* dataset. The overall procedure lasts roughly 150 min, including 20 to 30 min to operate the multimodal guiding system and allowed one to five distinct measurements localization.

### 2.7 Data Processing and Machine Learning Workflow

The data processing steps preceding the production of machine learning models involve extracting the Raman signature of the sample from the background signal, mostly associated with intrinsic tissue fluorescence. Raw data were averaged over the number of spectra (n), and cosmic ray artifacts were removed using a median filter. The calibration data acquired prior to the experiment were then used to subtract the dark count signal, normalize with the NIST standard measurement, and provide every spectrum with the x-axis Raman shift in ${\mathrm{cm}}^{-1}$. Finally, the background from autofluorescence signal was removed using a rolling ball algorithm, and the resulting Raman measurements were combined with labels (normal versus cancer) to be used as a training set input for the statistical analysis.

Due to the small size of the datasets, in comparison with the 1024 available intensity bins for each spectrum, the first step of the machine learning workflow consisted of dimensional reduction to <10 features. This dimensional reduction was achieved using a leave-one-patient-out cross validation (LOPOCV) random forest (RF) algorithm.^38^^,^^39^ Each feature was assigned a statistical weight quantifying its ability to capture interclass variations or “class purity,” allowing less important features to be dismissed. The remaining features were sorted according to their weight and a limited number of features associated with known prostatic molecular content was retained for model training. This feature selection process was applied independently twice: once on the raw 1024 feature set, and once again on the (<20) fitted Raman peaks extracted from the processed measurements. This method ensured a selection of <10 features notably linked to prostatic molecular content while still maintaining high statistical relevance for class separation.

The classification model training was an SVM with a linear kernel and a regularization parameter C. This hyperparameter, varying between $10^{-3}$ and 1, controlled the penalty for errors in the training process and prevented the model from overfitting since the number of observations used as support vectors remained <50% of the total observations. The SVM algorithm also considered the class imbalance (cancer versus normal) for all datasets by setting a higher cost γ to misclassified cancer spectra from normal spectra. A fixed weight value of 1 was set for the normal class, whereas γ varied between 0.2 and 5 for the cancer class. A grid search method was used to optimize both hyperparameters C and γ, and an LOPOCV procedure was performed to assess the classification model performance. The resulting model demonstrated optimal performance to discriminate cancer from normal tissue and returned for each measurement a posterior probability 0≤p≤1 to be classified as cancer. A receiver-operating-characteristic (ROC) curve was computed by comparing this posterior probability p with a parameter λ(0≤λ≤1). All observations associated by the classifier were assigned the label “cancer” when p≥λ or assigned the label “normal” if otherwise. Different values of the parameter λ corresponded to different points of the ROC curve. For all observations tested through cross validation, every point combines the comparison between the labels returned by the classifier and those returned by the histopathological gold standard to provide sensitivity and specificity values.

Results were reported for the optimized hyperparameters only, yielding the highest area-under-curve (AUC) value. The final optimized model, with reported accuracy, sensitivity, and specificity, corresponds to the ROC curve point with the smallest distance to the upper left corner of the curve.

## 3 Results

### 3.1 Spectroscopic Measurements

**Fig. 3:**
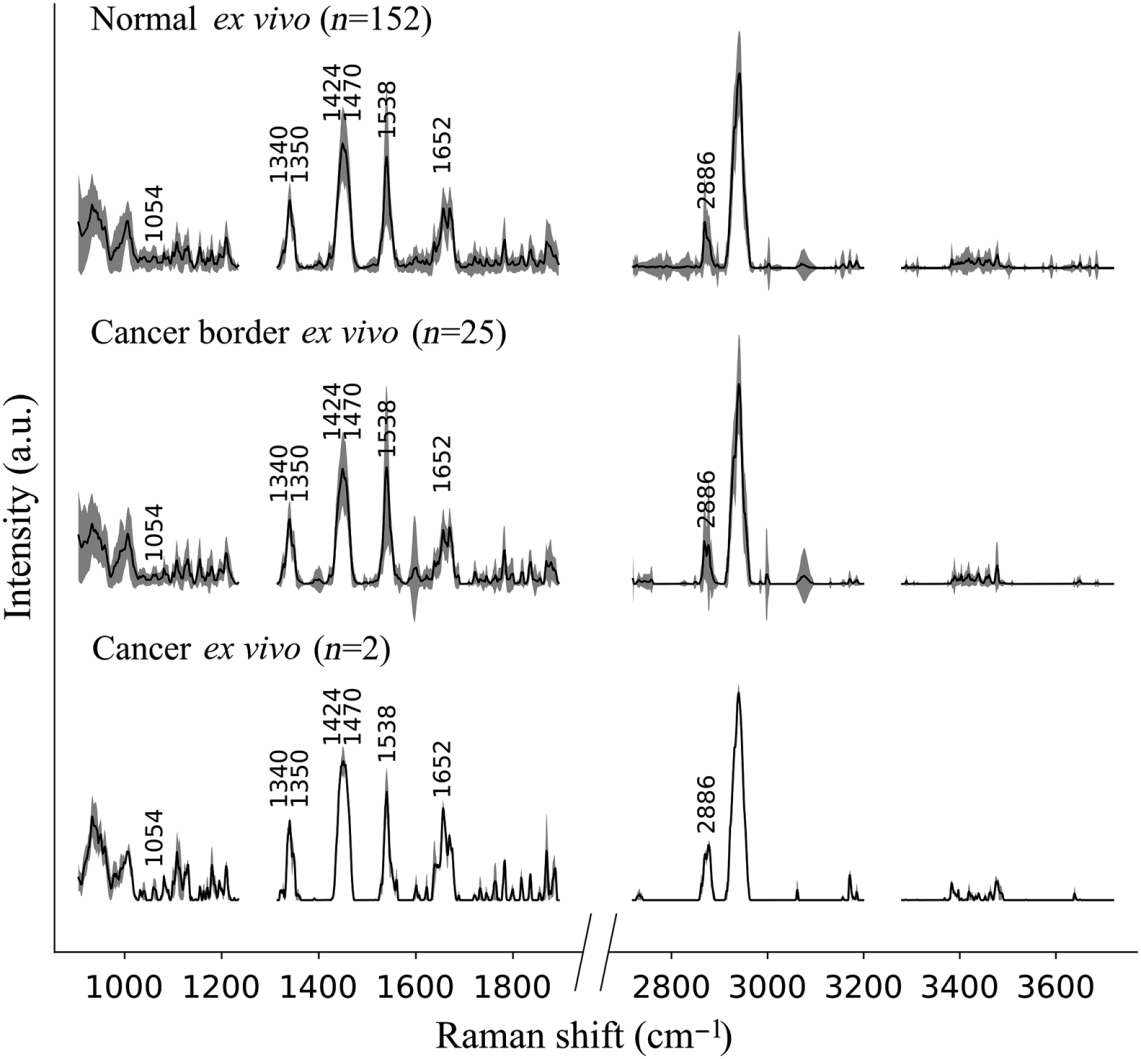
*Ex vivo* dataset: average and standard deviation of processed spectra for each category (normal, cancer border, cancer) with all peaks used by the classification models labeled with their Raman shift in ${\mathrm{cm}}^{-1}$.

The processed histopathological images of the 14 *ex vivo* prostate samples resulted in 179 spectroscopic measurements with colocated histopathology analyses, including 152 measurements labeled as normal prostate, 2 labeled as pure cancer, and 25 labeled as cancer border (mixture of normal prostate and cancer). A total of 20 additional measurements were located in normal tissue with cancer tissue within a <2.5  mm radius and were removed from the *ex vivo* dataset. Figure 3 shows the average and standard deviation for the Raman measurements of normal (n=152), cancer border (n=25), and cancer (n=2) for both FP (500 to $2000\;{\mathrm{cm}}^{-1}$) and HWN (2500- to $3800\;{\mathrm{cm}}^{-1}$) spectral domains. All three classes displayed similar high intensities for the phenylalanine peak at $1000\;{\mathrm{cm}}^{-1}$, the collagen peaks at 1340 and $1440\;{\mathrm{cm}}^{-1}$, the amides peaks at 1535 and $1650\;{\mathrm{cm}}^{-1}$, and the lipids and protein peaks at 2880 and $2930\;{\mathrm{cm}}^{-1}$. Qualitative assessment of the spectral bands did not allow direct discrimination between classes. However, subtle differences in peak intensities remained noticeable between classes by qualitative visual inspection. For instance, the intensities of the HWN lipids peak doublet at $2880/2886\;{\mathrm{cm}}^{-1}$ shifts from right to left when comparing normal to cancer, with cancer border showing even intensity of the two peaks. Similarly, the amide peak at $1650\;{\mathrm{cm}}^{-1}$ was stronger for the cancer class but was identical between the cancer border and normal classes.

**Fig. 4:**
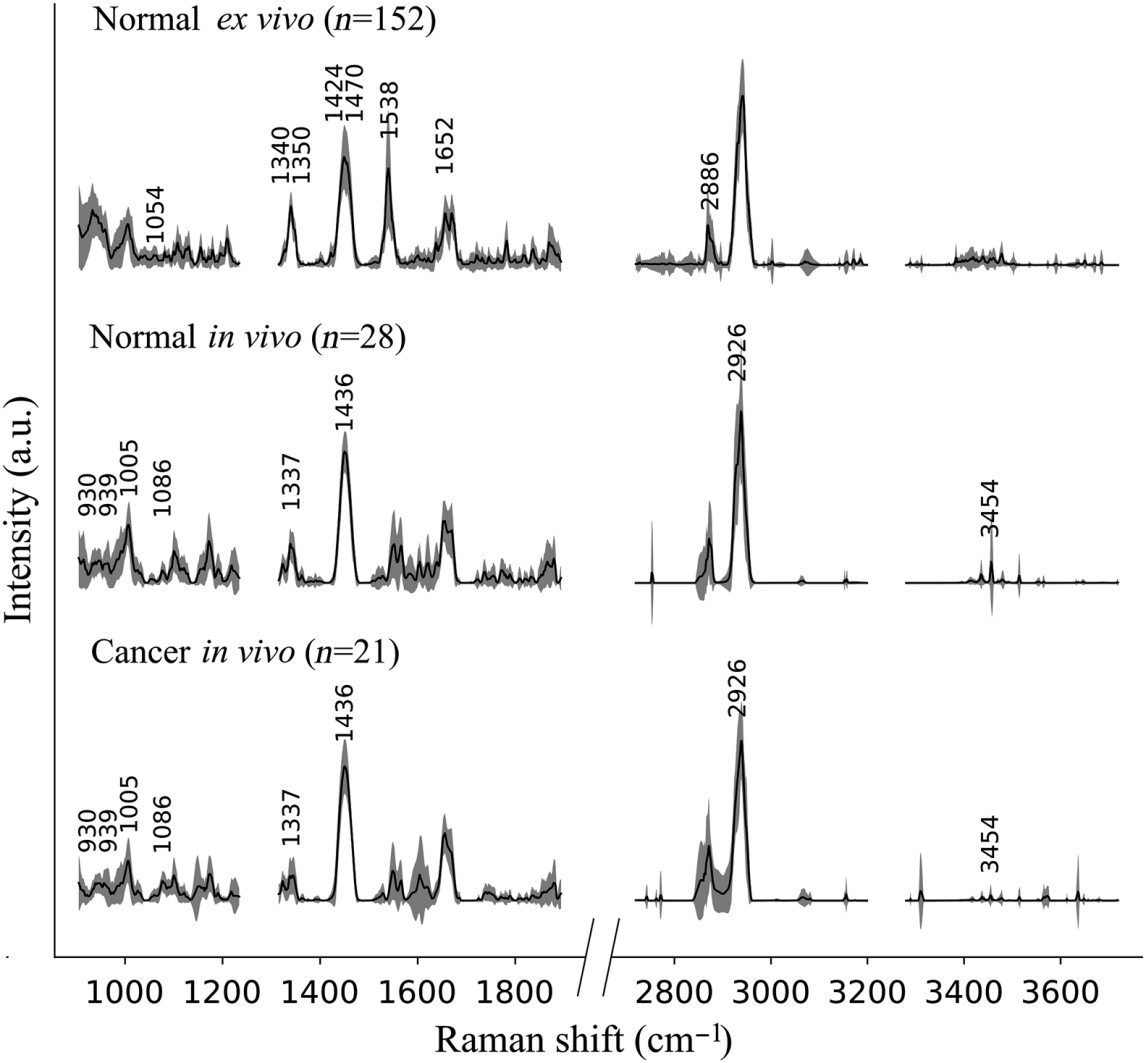
Average and standard deviation of processed spectra for normal and cancer *in vivo* spectra with all peaks used by the classification models labeled with their Raman shift in ${\mathrm{cm}}^{-1}$. The *ex vivo* spectra from normal prostate are shown for comparison.

The *in vivo* acquisition from 18 patients led to 49 RS measurements, including 28 labeled as normal and 21 labeled as cancer. Figure 4 shows the average and standard deviation of these Raman measurements for FP (500 to $2000\;{\mathrm{cm}}^{-1}$) and HWN (2500 to $3800\;{\mathrm{cm}}^{-1}$) spectral domains. Also shown are the average and standard deviation of the 152 *ex vivo* Raman measurements labeled as normal prostate tissue to allow a spectra-to-spectra comparison of the biomolecular content of *ex vivo* normal tissue and *in vivo* spectra. Similarly to the *ex vivo* measurements, the *in vivo* spectra displayed the phenylalanine peak at $1000\;{\mathrm{cm}}^{-1}$, collagen peaks at $1335/1440\;{\mathrm{cm}}^{-1}$, and amides peaks at $1535/1650\;{\mathrm{cm}}^{-1}$ in the FP region, as well as the lipids and protein peaks at $2880/2930\;{\mathrm{cm}}^{-1}$ in the HWN region. Although these spectral bands did not allow for strict discrimination between *in vivo* cancer and *in vivo* normal classes, slight differences remained visible. The phenylalanine and collagen peaks, at 1000 and $1335\;{\mathrm{cm}}^{-1}$, respectively, displayed higher intensity in normal measurements than in cancer, and an additional peak at $3454\;{\mathrm{cm}}^{-1}$ (O–H molecular bond) also displayed higher intensity in normal than in cancer.

Lastly, we identified one major difference between *ex vivo* normal spectra and *in vivo* spectra for the amide peak at $1535\;{\mathrm{cm}}^{-1}$ only. Figure 4 shows an intensity that is >3 times greater for *ex vivo* normal than it is for both *in vivo* spectra (normal and cancer).

### 3.2 Machine Learning Cancer Detection Models

The classification model trained on the 179 *ex vivo* Raman measurements yielded an accuracy of 67%, a sensitivity of 63%, and a specificity of 68%. This optimized statistical model was obtained with an ROC curve with an AUC of 0.72 and used eight spectral features. The classification model trained on the 49 *in vivo* Raman measurements yielded an accuracy of 79%, a sensitivity of 86%, and a specificity of 72%. This optimized statistical model was obtained with an ROC curve with an AUC of 0.77 and used eight features.

**Fig. 5:**
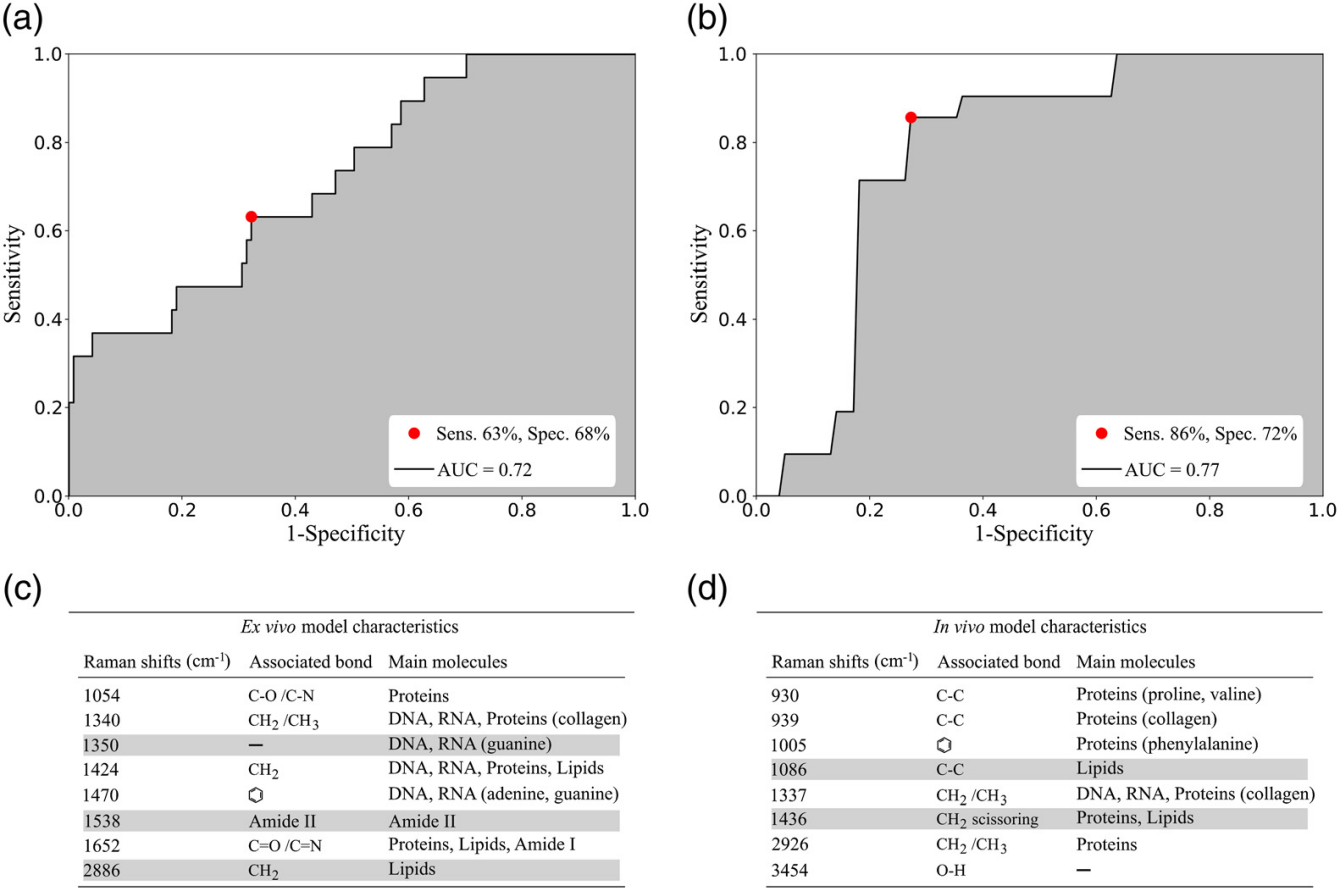
ROC curve for discriminating normal from cancer prostatic tissue using RS: (a) *ex vivo* model and (b) *in vivo* model. List of Raman features associated with prostatic Raman-predicted molecular content that are used as inputs for the classification models: (c) *ex vivo* and (d) *in vivo*. Features with Raman band intensities higher in the cancer class are highlighted in gray. The molecular assignment of Raman peaks was based on literature findings.^29^^–^^31^^,^^40^

These classification results between cancer and normal tissue are presented as ROC curves for the *ex vivo* and *in vivo* datasets in Figs. 5(a) and 5(b), respectively. In addition, Figs. 5(c) and 5(d) list the Raman features used for both models and their molecular assignment based on the literature.^29^^–^^31^^,^^40^ Moreover, the *ex vivo* and *in vivo* models used DNA, RNA, proteins, and lipids features. Amides, adenine, and guanine at $1350/1470/1538/1652\;{\mathrm{cm}}^{-1}$ served the *ex vivo* model only, whereas the phenylalanine at $∼1000\;{\mathrm{cm}}^{-1}$ was used only for the *in vivo* model. Finally, the collagen peak at $1335\;{\mathrm{cm}}^{-1}$ was a feature in both models.

## 4 Discussion

Although limited in their accuracy, the classification models built from the *ex vivo* and *in vivo* datasets demonstrate that RS could discriminate PCa from normal tissue in a laboratory environment or *in situ* during surgery. On *in vivo* specimens, the spectra of normal and PCa showed differences in peaks at 1086 and $1436\;{\mathrm{cm}}^{-1}$ [Fig. 5(c)] while the *ex vivo* spectra showed differences at $2886\;{\mathrm{cm}}^{-1}$ [Fig. 5(d)]. These differences are indicating the presence of greater amounts of lipids in cancer locations. The presence of lipids peaks has also been demonstrated in a study of PCa by Medipally et al.^41^ in which they found an alteration of lipid metabolism. Moreover, in this study, the bands corresponding to lipids were more abundant in the plasma of PCa patients compared to healthy individuals. Indeed, due to their high proliferation rate, cancer cells depend upon a large amount of metabolic energy to synthesize cellular membranes.^42^ Lipids in cancer cells are therefore the main fuel source for membrane synthesis and become one of the major actors in tumor cells growth.

Furthermore, in the *ex vivo* specimens, the peak at $1350\;{\mathrm{cm}}^{-1}$ assigned to DNA and RNA (guanine) is higher in intensity in cancerous prostate tissues than healthy tissues [Fig. 5(d)]. This is consistent with many studies that have demonstrated genetic dysregulation in PCa.^43^^–^^46^ In a study by Gao et al.,^47^ they found that aggressive PCa has the guanine allele- and thus, this allele was linked to higher expression of genes that are tied to the growth of cells and progression of tumors in PCa.

The peak at $1538\;{\mathrm{cm}}^{-1}$ assigned to the amide group is also higher in PCa patients, which implies an increase of protein concentration, and is consistent with the literature that found higher amide peak in breast cancer,^48^ lung cancer,^49^ and colorectal cancer tissues.^50^ One of the proposed explanations for this difference is that it appears to inform on biological changes of malignancy such as increased alterations in protein structures and protein unfolding.

A limitation of the study is the small size of the datasets from which the classifiers were built. However, to minimize the likelihood of overfitting, we constrained the training process to using <10 features while the dataset size was at least five times larger. We also ensured—through qualitative inspection of the spectra—biomolecular interpretability of the features since the risk of overfitting rises with the use of features that are purely associated with statistical variance between observations. Another limitation relates to the limited reliability associated with the gold standard of histology used to assign labels to each *ex vivo* measurement. The HPS staining used for histopathological analysis resulted in registration errors between Raman measurements of localization and the reconstructed prostates slices used to assign tissue labels (cancer versus normal prostate). This limitation was mitigated for the measurements labeled as normal tissue in which a high margin of error criterium (2.5-mm radius confidence area) kept 152 normal measurements in the dataset but led to the removal of 20 measurements. However, when the same criterium, plus one that was more lenient (1.25-mm radius confidence area), was applied to measurements labeled as cancer, only 2 cancer and 25 cancer border measurements were retained. As a result, the cancer class, summing up to 27 measurements for the *ex vivo* dataset, showed not only a low number of measurements but was also at risk of containing mixed cancer and normal labels. These two major limitations of the *ex vivo* dataset present a plausible explanation of the underperformance of the resulting model in comparison with the classifiers trained with the *in vivo* dataset, which was limited only by its small sample size. Due to the small biopsy sample size for the *in vivo* dataset and the highly accurate colocation with the RS measurements, the gold standard (histology) accuracy was not limited by the spatial registration error between optical measurements and the sample’s location.

To overcome the pitfalls of small datasets, a possible solution was to merge *ex vivo* and *in vivo* measurements. The spectra-to-spectra comparison highlighted their compatibility in terms of identified prostatic molecular contents (e.g., amides I, collagen, phenylalanine). The only notable exception was the $1535\;{\mathrm{cm}}^{-1}$ peak related to amide II, which was identified in all measurements but displayed an average intensity that was three times greater in the *ex vivo* measurements. However, merging of the two datasets solution was not used for this study due to the low reliability of the histology for the *ex vivo* dataset. Furthermore, this approach would require accounting for signal changes associated with temperature-induced optical properties variations. For example, Ghita et al. demonstrated that differences in temperature >20°C can affect the intensity of the collected signal and lead to Raman spectra changes.^51^

Future efforts should focus on increasing the amount of data by increasing the number of recruited patients. The number of measurements obtained per patient was consistently less than four, but the acquisition time did not require more than 20% of the total experiment duration. Combining the robustness of our *in vivo* dataset acquisition method with a larger patient cohort would be the next step in evaluating PCa detection using our multimodal method based on the RS system. Future work should also expand to other clinical applications with the same issue of tumor localization such as brachytherapy procedures. Indeed, the treatment procedure efficiency relies on the accurate radiation delivery dose on the tumor while minimizing healthy tissue exposition, and an assisting real-time Raman system would significantly improve the tumor outline detection. However, to avoid any registration problems caused by extra-cannulas insertion for the Raman probe, the tumor detection would need to be performed directly through the brachytherapy plastic guiding cannulas. This detection through a plastic layer has precedents and literature indicates that spatial offset Raman spectroscopy could achieve it by providing depth resolution to the collected measurements.^52^

## Code, Data, and Materials Availability

The data and materials information that support the findings of this study are available from the corresponding author upon reasonable request.
